# Cross-section of thyroidology and nephrology: Literature review and key points for clinicians

**DOI:** 10.1016/j.jcte.2024.100359

**Published:** 2024-07-07

**Authors:** Joe M. Chehade, Heiba F. Belal

**Affiliations:** Division of Endocrinology, Diabetes, and Metabolism, Department of Medicine, University of Florida Jacksonville College of Medicine, FL, USA

## Abstract

There are several key points clinicians should consider when managing patients with overlapping thyroid and renal disease. Patients who are euthyroid and have chronic kidney disease (CKD) may physiologically have normal-high thyroid stimulating hormone (TSH), low free thyroxine (FT4), low free triiodothyronine (FT3) and normal-low reverse triiodothyronine (rT3). Untreated subclinical and primary hypothyroidism among patients with (CKD) is associated with reversible progression of renal failure. Supplementing these (CKD) patientswith levothyroxine can delay the progression of renal failure and prevent end stage renal disease (ESRD). Untreated hyperthyroidism increases the glomerular filtration rate (GFR) by 18 to 25%. Thus, the management of hyperthyroidism may unmask patients with undiagnosed CKD. There is no dosage adjustment required for methimazole among patients with CKD. However, methimazole may be eliminated during hemodialysis (HD) by around 30 to 40%. Patients with papillary thyroid cancer and ESRD may have higher rates of aggressive characteristics. Patients with CKD and ESRD undergoing radioiodine I-131 treatment for thyroid cancer are at increased risk of prolonged radiation transmission risk due to decreased iodine urinary excretion. Additionally, the optimal dosing and timing of radioiodine I-131 therapy amongst patients with ESRD and thyroid cancer requires further research. The use dosimetry studies and multidisciplinary coordination among nuclear medicine, nephrology and endocrinology is recommended for these patients.

## Introduction

Chronic kidney disease (CKD) affects one in seven adults in the United States. Similarly, more than 12 % of adults in the United States will develop a thyroid condition in their lifetime. Given such common prevalence of overlapping thyroid and renal disorders, a substantial patient population is at risk. These patients require individualized care and thoughtful attention towards the interplay of thyroid and renal disease. This review will highlight key points for clinicians regarding the impact intersecting thyroid and renal disorders have on pathophysiology, clinical presentation as well as diagnostic, prognostic, and management decisions.

Thyroid hormone (TH) production and regulation are managed by the hypothalamus-pituitary-thyroid axis. The hypothalamus produces thyroid releasing hormone (TRH) which stimulates the anterior pituitary to produce thyroid stimulating hormone (TSH) and further thyroxine (T4) and triiodothyronine (T3) production from the thyroid gland. Most of the iodine excretion occurs through the urinary system, with only a small amount excreted through the gastrointestinal tract. Renal failure and the resulting state of chronic metabolic acidosis, hypoalbuminemia and uremia impacts TH production, regulation, metabolism as well as iodine excretion.

The connection between thyroid and kidney function has been a well-known phenomenon for many decades [1]. Thyroid hormones play a crucial role in adequate renal growth and development. Additionally, both overactive and underactive thyroid conditions are associated with significant changes in water and electrolyte metabolism which influence cardiovascular function and thereby renal function.

This current review will summarize the following key clinical considerations among patients with chronic renal failure (defined as decreased estimated glomerular filtration rate (eGFR) < 60 ml/min/1.73 m^2^ over at least 3 months irrespective of etiology): 1) expected alterations in TH production, regulation and iodine excretion 2) impact of hyper- and hypothyroidism on renal failure outcomes 3) management and surveillance of thyroid cancer and 4) dosing, timing and efficacy radio-iodine ablation therapy.

## Thyroid function & nephrology

### Thyroid releasing hormone & thyroid stimulating hormone

The response of TSH to exogenous TRH is blunted among patients with end-stage renal disease (ESRD) who are euthyroid both before and after dialysis treatment [1]. This may be related to elevated peak levels, prolonged half-life, and reduced clearance of exogenous TRH among patients with ESRD.

Patients with ESRD exhibit alterations in the physiological circadian rhythm of pulsatile TSH secretion. The natural peak in evening TSH and well as the TSH pulse amplitude are both diminished in these patients [2]. TSH clearance rates have been shown to be reduced by 57 % among patients with ESRD, which may be related to reduced renal clearance [3]. Fortunately, these changes do not impact T4 production among patients with ESRD and they maintain normal steady state.

Similarly, patients with severe non-thyroidal illnesses, TSH glycosylation is altered in euthyroid patients with ESRD [1]. This may change the plasma half-life and bioactivity of TSH. Additionally, TSH alpha-subunit levels are significantly higher in patient who are euthyroid with ESRD compared to healthy subjects who are euthyroid [4].

### Thyroxine

Approximately 99.98 % of T4 is bound to T4 binding proteins: thyroxine-binding globulin (TBG), transthyretin, albumin, and lipoproteins. Among patients with CKD, low albumin levels contribute to low total thyroid hormone and low free thyroid hormone [5].

Thyroxine analog assays are used routinely in clinical practice. These assays indirectly estimate the exact fraction of non-protein bound T4 [6]. The thyroxine analog assay relies on free thyroxine (FT4) protein binding. In patients with ESRD, FT4 analog assays may be falsely low in the setting of hypoalbuminemia. A direct FT4 assay can be used to directly determine circulating FT4 levels. This approach physically separates free- from protein- bound T4 using ultrafiltration or equilibrium dialysis [7]. This step is then followed by measurement of free hormones using radioimmunoassay or liquid chromatography/tandem mass spectrometry.

In addition to hypoalbuminemia, uremia can also affect the accuracy of FT4 assays producing falsely low levels [6]. Assays that can produce falsely low T4 levels among renal failure patients include immunophase and liquisol methods [1]. Considering these factors, FT4 by dialysis assay serves as a preferred methodology to accurately assess T4 levels among patients with CKD and ESRD [6].

Heparin administration during hemodialysis (HD) can cause false elevations in total T4 and FT4 Levels [8]. A rise in FT4 concentrations up to 5-fold at 2 to 15 min due to the generation of non-esterified fatty acids have been reported. This artifact has been detected with different assays, including direct immunoassays, ultracentrifugation, and equilibrium dialysis. We recommend measuring a blood sample at least 10 h after the last heparin administration.

### Triiodothyronine

The most frequent laboratory abnormality among patients with CKD and ESRD is low T3, regardless of normal TSH levels [9]. In a study evaluating 287 patients with normal thyroid function and ESRD, 76 % had total T3 levels under 100 ng/dl, and 65.5 % had free T3 index levels lower than 100 [10]. The reported decline in T3 levels is due to reduced peripheral tissue conversion of T4 to T3. Nevertheless, production of T3 by the thyroid gland remains normal and T3 clearance rates are decreased or normal, as seen in other non-thyroidal illnesses.

Most of the T4-to-T3 conversion occurs in the liver via type 1 and type 2 deiodinases. However, a significant amount of T4 to T3 activation also happens in the kidneys and can be reduced by the presence of renal failure. Additionally, chronic metabolic acidosis can negatively impact T4 deiodination and lower overall T3 levels [5]. Of note, low T3 levels among ESRD patients are an independent predictor of mortality [11]. Additionally, a retrospective study of 2,284 euthyroid cases found a positive relationship of eGFR with serum T3 levels independent of age and serum albumin. An eGFR < 60 ml/min/1.73 m^2^ was associated with an increased odd of low T3 [Odds ratio 2.40 (confidence interval (CI): 1.5315–3.1731)] [9]. Despite low T3 levels among patient with ESRD, most maintain euthyroidism [1]. Low T3 levels among patients with CKD may contribute to reducing protein catabolism and thereby producing less nitrogen accumulation [12], [13]. Clinician should consider this potential adverse outcome when deciding whether to treat asymptomatic patients with CKD and low T3 levels. Currently there is no clinical evidence for improved renal outcomes with levothyroxine supplementation among patients with CKD and low T3 levels [14].

### Reverse triiodothyronine

Type 3 deiodinase catalyzes the conversion of T4 into reverse T3 (rT3) and then into diiodothyronine. Contrary to patients with euthyroid sick syndrome who have elevated rT3 levels, euthyroid patients with CKD have normal or low rT3 levels [12]. Additionally euthyroid patients with CKD tend to have elevated TSH levels [1] as opposed to lower TSH commonly seen in patients with euthyroid sick syndrome (Table 1). Interestingly, the severity of albuminuria among patient with CKD, independent of renal function, is negatively correlated with rT3 levels; nephrotic range albuminuria exhibits lower rT3 [12].

**Table 1:** .

	**Non-Thyroidal Illness** **(Aka Euthyroid Sick Syndrome)**	**CKD/Uremia**	**Primary Hypothyroidism**
**TSH**	Normal or	Normal or	
**FT4**			
**FT3**			Normal or
**rT3**		Normal or	
**Goiter**	None	Higher Chance	+/-

### Iodine

In advanced CKD, iodine accumulates due to reduced renal excretion. This prolonged thyroid exposure to iodine can block thyroid hormone production via the Wolff-Chaikoff effect [1]. Prolonged iodine exposure has also been linked to an increased incidence of goiter among CKD patients [13]. In vitro thyroid function was studied among 30 patients undergoing hemodialysis (HD), using perchlorate (inhibits iodine transport into thyroid) discharge testing and I-131 salivary to plasma ratio, in order to identify defects in iodine uptake and organification [15]. Compared to 35 healthy controls, patients undergoing hemodialysis had lower T3, lower T4 uptake and higher T3 resin uptake, indicating impaired iodine trapping [15]. Additionally, patients undergoing HD had higher radio-iodine uptake one hour after perchlorate, indicating enhanced activity of the thyroid ATP-dependent iodine pump. Despite these findings, TSH levels did not differ between the two groups, indicating normal organification among patients undergoing HD.

### Nephrotic syndrome

In a cross-section study of 31 pediatric patients with nephrotic syndrome (NS) and stable eGFR and creatinine, 51.6 % had abnormal thyroid tests including low total T3, total T4 and free T4 [16]. Interestingly, the severity of proteinuria at initial NS onset, measured by urinary protein to creatinine ratio, correlated with severity of serum T3, T4, TSH, and FT4 alterations. Additionally, serum albumin levels correlated with T3 levels. The mechanism of thyroid dysfunction in NS is hypothesized to be related to increased urinary loss of free protein-unbound and protein-bound thyroid hormones. This hypothesis is supported by the impromptu return of euthyroidism alongside remission of proteinuria. Additional factors which may play a role in the pathogenesis of thyroid dysfunction in NS include NS treatment duration, type of immunosuppressive agents, altered T4-to-T3 conversion ability and compensatory thyroid mechanisms in response to NS. There is limited evidence regarding the need for levothyroxine administration in hypothyroidism due to NS. However, it is well established that in patients with non-NS related hypothyroidism and concurrent NS the levothyroxine dose must be increased.

## Key points

Patients with ESRD may have falsely low FT4 levels when using analog assays due to hypoalbuminemia and uremia.
  Utilization of direct FT4 assays such as ultrafiltration of equilibrium dialysis is recommended in clinical scenarios where FT4 levels do not correlate with the clinical presentation among patients with CKD/ESRD.
Low FT3 is the most frequent thyroid abnormality found amongst patients with CKD /ESRD
  Low FT3 is correlated with increased mortality among patients with ESRD.
  There is limited data regarding clinical benefit of levothyroxine supplementation amongst euthyroid patients with CKD/ESRD and low T3.
There are several keyways to differentiate non-thyroidal illness from euthyroid patient with CKD/ESRD:
  TSH tends to be low in non-thyroidal illness and normal-high in CKD/ESRD.
  rT3 tends to be high in non-thyroidal illness and normal-low in CKD/ESRD.
Patients with CKD/ESRD exhibit altered thyroid iodine trapping. However, organification remains normal.
Decreased urinary iodine excretion in patients with CKD/ESRD results in increased iodine exposure which can contribute to an increased incidence of goiter among these patients.

## Hypothyroidism & nephrology

The presence of CKD is linked with an increased incidence of non-autoimmune subclinical and primary hypothyroidism. The National Health and Nutrition Examination Survey revealed that hypothyroidism occurred in 10.9 % of patients with stage 2 CKD, 21.0 % of patients with stage 3 CKD, and 23.1 % of patients with stage 4 or 5 CKD [18]. In these patients with hypothyroidism, 56 % had subclinical hypothyroidism. A cross-sectional analysis of 3089 patients found the prevalence of subclinical hypothyroidism to be 18 % among patient with CKD not requiring dialysis [19]. Furthermore, patients with GFR < 30 ml/min/1.73 m^2^ are twice as likely to develop hypothyroidism compared to patients with GFR > 90 ml/min/1.73 m^2^ [20]. There is an inverse association between eGFR and risk of hypothyroidism independent of age, sex, race, or co-morbidity status [19]. There is currently no proven causal relationship for the association between hypothyroidism and CKD.

Mounting evidence indicates that hypothyroidism is likely linked with higher risk of cardiovascular disease, adverse patient outcomes, and decreased survival among the advanced CKD and ESRD population [20]. Among patients with CKD, hypothyroidism is associated with higher all-cause mortality irrespective of dialysis status, socio-demographics, or co-morbidities [5]. Nonetheless, subclinical hypothyroidism has been associated with lower left ventricular ejection fraction among patients undergoing peritoneal dialysis even when adjusted for age, diabetes, and previous coronary artery disease [21]. Several studies have shown a positive correlation between higher TSH levels and higher coronary artery calcification among patientswith ESRD [22].

There is currently limited data exploring treatment of hypothyroidism to suggest any benefits among patients with CKD/ESRD [20]. However, untreated subclinical and primary hypothyroidism are both associated with a reversible increase in serum creatinine and decrease in eGFR [5]. There are several proposed mechanisms for this association. Animal studies have shown that hypothyroidism adversely affects kidney size, development and structure [5]. Furthermore, hypothyroidism has been suggested to impair the renin-angiotensin-aldosterone system (RAAS) and thereby alter renal perfusion [5]. The link between hypothyroidism and decreased expression of renal tubular ion transporters (i.e. Na-K-ATPase) has also been shown to result in increased tubulo-glomerular feedback [5]. Additionally, renal perfusion is delayed in hypothyroidism due to reduced cardiac output, decreased renal vasodilator production, and decreased red blood cell production [5].

A bi-directional mechanism has been proposed as the causation of hypothyroidism among patients with CKD. High iodine retention may induce a Wolff-Chaikoff effect and thereby increase the risk of hypothyroidism in CKD [23]. Metabolic acidosis among patientswith CKD has also been shown to increase TSH and decrease FT4 and FT3 [24]. Finally, selenium deficiency, malnutrition and protein loss may also serve as mechanistic links for the development of hypothyroidism among patients with CKD [23].

There are several studies that show reno-protective benefits when treating both subclinical and primary hypothyroidism among patients with CKD. A retrospective study of 309 patients with CKD stage 2–4 and subclinical hypothyroidism (excluding those with TSH greater than 10 μIU/ml) found that the rate of decline in eGFR was significantly greater in untreated patients compared to those treated with levothyroxine [25]. During the mean follow up of 34.8 ± 24.3 months, a 50 % decrease in eGFR occurred in 20.9 % of the untreated group and in only 8.3 % of the treated group. The incidence of ESRD was higher in the non-treatment group compared to the treatment group (2.36 vs*.* 0.37 per 100 patients-years; *P =* 0.01). This study showed that thyroid hormone replacement in subclinical hypothyroidism among patients with CKD resulted in risk reduction for halving eGFR of 64 % and of 85 % for decreasing development of ESRD. Finally, the renal event-free survival was significantly lower in those not treated with levothyroxine. The beneficial impact of thyroid hormone replacement remained significant even after adjustment for age, sex, diabetes mellitus status, eGFR stage and lipid profile. The authors hypothesized that improved cardiac dysfunction, endothelial dysfunction, and dyslipidemia may be responsible for preserved renal function among patients with CKD treated for subclinical hypothyroidism.

## Key points

There is an increased incidence of non-autoimmune subclinical and primary hypothyroidism among patients with CKD/ESRD compared to patients with normal renal function.
Possible bi-directional mechanisms for the development of hypothyroidism among patients with CKD/ESRD include:
  Altered kidney development and structure [20].
  Impaired RAAS and renal perfusion [5].
  Decreased expression of renal tubular ion transporters (i.e., Na-K-ATPase) resulting in increased tubulo-glomerular feedback [5].
  Delayed renal perfusion is in hypothyroidism due to reduced cardiac output, decreased renal vasodilator production, and decreased red blood cell production [5].
Untreated subclinical and primary hypothyroidism are both associated with reversible worsening progression of renal failure amongst patients with CKD [5].
Administration of levothyroxine in patients with CKD and subclinical hypothyroidism has been shown to delay the progression of renal failure and possibly prevent ESRD [25].

## Hyperthyroidism & nephrology

The Jod-Basedow phenomenon and iodine retention in ESRD have been hypothesized to contribute to hyperthyroidism among patients with ESRD [22]. However, there are only a few case reports of iodine-induced hyperthyroidism amongst patients with ESRD [5]. A review of fourteen cases of hyperthyroidism in ESRD found that one third of patients developed hyperthyroidism within one year of HD initiation [26]. Nonetheless, the prevalence of hyperthyroidism amid patients with CKD and ESRD is comparable to the general population.

Hyperthyroidism impacts several physiological processes which influence key renal parameters. Elevated THs act to decrease systemic vascular resistance, increase cardiac output, increase endothelial nitric oxide production (thyroid hormone directly induces nitric oxide synthase), increase renal β-1 adrenergic receptor activation, increase macula dense sensitivity, increase RAAS activation, increase renin gene expression directly by T3, increase angiotensin synthesis by liver, increase tubulo-glomerular feedback, increase renal blood flow, and increase Na-K-ATPase activity [27]. Through these mechanisms hyperthyroidism increases GFR in patients by up to 18 to 25 % [27]. Serum creatinine thereby is decreased in untreated hyperthyroidism. This decrease in creatinine is compounded by the decreased muscle mass present in hyperthyroidism. The increase in GFR in patients with hyperthyroidism is reversed once euthyroidism is achieved with adequate treatment. Thus, treatment of hyperthyroidism may unmask patients with underlying renal failure.

Renal failure can progress rapidly in cases of untreated hyperthyroidism. Several mechanisms are responsible for this mechanism [13]. The increased renal blood blow in hyperthyroidism causes intra-glomerular hypertension and hyper-filtration. This increased filtration pressure can produce proteinuria which synergistically causes direct renal injury. Additionally, hyperthyroidism increases mitochondrial energy metabolism while also down regulating superoxide dismutase. These processes contribute to the increased presence of free radicals in hyperthyroidism which directly accelerates renal failure. Indirectly, oxidative stress in thyrotoxicosis can contribute to hypertension which thereby progresses renal disease. In addition, hyperthyroidism and the increased RAAS state contribute to renal fibrosis and worsening glomerulosclerosis [28]. Untreated hyperthyroidism accelerates CKD progression significantly due to these pathophysiologic processes and requires prompt identification and management.

Antithyroid medications are the mainstay in the management of thyrotoxicosis. Methimazole is metabolized by the liver and excreted by the kidney. There are no methimazole dosage adjustments required for patients with CKD. However, there are key considerations when using methimazole among patients with ESRD. Komine et al. investigated the pharmacokinetics of methimazole among patients with ESRD on non-dialysis days as well as its dialyzability [26]. They found that the serum half-life of methimazole amongst patients with ESRD is similar to that in patients with normal kidney function. Furthermore, they determined that methimazole was eliminated during HD by around 30–40 % [26]. However, their article cites several similar studies with conflicting results, concluding there may be significant inter-individual differences in the absorption of methimazole among patients with CKD. More research is needed to assess the intra-thyroidal turnover of methimazole amongst patients with uremia.

In regard to using radioiodine 131 ablation to treat hyperthyroidism amongst patients with ESRD, important considerations should be made. Inorganic iodine is cleared by dialysis. Thus, serum iodine levels are lowest immediately after dialysis. To optimize radioiodine 131 uptake and ablation success, some recommend performing dialysis immediately before administration of radioiodine 131 [29]. These authors recommend waiting at least ten hours after the radioiodine 131 dose before performing dialysis. Furthermore, they recommend no adjustments in radioiodine 131 dosing when treating hyperthyroidism among patients with ESRD. However, amongst patients with CKD and hyperthyroidism, some recommend using lower doses of radioiodine 131 [27]. Further research is required to determine the optimal management of radioiodine 131 therapy amongst patients with hyperthyroidism and CKD/ESRD.

## Key points

The prevalence of hyperthyroidism amongst patients with CKD and ESRD is comparable to the general population.
GFR is increased by around 18–25 % among patients with hyperthyroidism [27]. Adequately treating hyperthyroidism may unmask patients with underlying abnormal renal function.
Untreated hyperthyroidism accelerates CKD progression [28].^.^
There are no methimazole dosage adjustments required for patients with CKD.
There is some evidence that methimazole may be eliminated during hemodialysis by around 30–40 % [26]. Further research is needed to establish the metabolism and excretion of methimazole among patients with CKD/ESRD.
Further research is required to determine the optimal management of radioiodine 131 therapy amongst patients with hyperthyroidism and CKD/ESRD.

## Thyroid Nodules, thyroid cancer, and renal cancer

Thyroid and renal cancer are two distinct malignancies, each with their own unique epidemiology, risk factors, and treatment approaches. However, there are instances where these two cancers can coexist in a patient, either as separate primary malignancies or as a result of metastasis. Thyroid carcinoma is a polygenic disease which may be associated with other malignancies. In terms of risk factors, both thyroid and renal cancers have been associated with certain genetic syndromes. However, most cases of thyroid and renal cancers are sporadic.

The effect of external irradiation and radioiodvermaine 131 treatments are well established to have potential carcinogenic effects. Following treatment of differentiated thyroid cancer observational studies have confirmed an increased risk of developing secondary cancers [30], [31]. In one study after a median follow-up of eight years, women with differentiated thyroid carcinoma were at higher risk of developing a second cancer of the genitourinary tract and kidney [31].

Subramanian et al., in a systematic review and *meta*-analysis, examined the standardized incidence ratios of second primary malignancies amongst thyroid cancer survivors in comparison to individuals without thyroid cancer [30]. They demonstrated that thyroid cancer survivors are at increased risk of second primary malignancies. This may be linked to disease-specific treatments or genetic predispositions. The standardized incidence ratios of the ensuing second primary malignancies of the salivary gland, stomach, colon/colorectal, breast, prostate, kidney, brain/central nervous system, soft tissue sarcoma, non-Hodgkin's lymphoma, multiple myeloma, leukemia, bone/joints, and adrenal were all significantly increased.

The thyroid gland can be involved by metastases from renal, colorectal, lung and breast carcinomas, and melanomas [32]. These metastases are hardly ever linked to any thyroid dysfunction.

Most patients with thyroid cancer (intermediate and high risk) are managed with total-thyroidectomy followed by radioiodine 131 remnant ablation and active biochemical and imaging surveillance. Overall rates of thyroid cancer among patients with CKD and ESRD are similar to the overall population [33]. However, in a recent retrospective review, papillary thyroid cancer (PTC) was found to be more common among patients with ESRD compared to normal controls [34]. This study noted that PTC cases in the ESRD group had a significantly higher rate of aggressive characteristics such as capsule invasion, multi-focality, and lymph node metastasis.

Chronic kidney failure may increase the risk of renal cancer by several mechanisms, such as the induction of inflammation and accumulation of uremic toxins in non-functioning kidneys [35]. Kidney cancer and thyroid cancer demonstrate higher occurrences during dialysis intervals and lower occurrences during functional kidney periods post-transplant [35]. Comparable results were noticed in a cohort of kidney transplant recipients in Australia and New Zealand [36], [37]. Among solid organ transplant recipients compared to the general population, Kitahara et al. found a 2.5-fold increase in thyroid cancer incidence [38]. Kidney organ recipients had the highest incidence of thyroid cancer compared to other recipients of different organs [38].

Regarding thyroid nodules among patients with ESRD, the prevalence is higher compared to control subjects with normal renal function. In one study, the prevalence of thyroid nodules amongst female patients on hemodialysis was 55 % compared to 21 % amongst controlled females with normal renal function [39]. Da Costa et al. reported that patients with ESRD had more hypoechoic nodules compared with controls (24.1 % vs. 7.9 %, P = 0.056) [40]. A large retrospective Italian cohort study of 2,147 patients with ESRD on the renal transplant waiting list examined a group of 437 with hypothyroidism and demonstrated that 45.8 % had a nodular disease [41]. A single benign thyroid nodule was found among 60 %. Goiter was found among 26.5 %; neoplasm was discovered among 13.5 %.

Finally, the impact of thyroid cancer treatment on renal function should be considered. Di Paola et al. reviewed the literature to highlight how different therapeutic approaches impact renal function among patients with renal failure [42]. One consideration is that there is a higher incidence of acute kidney injury after total thyroidectomy. Another point is that pharmacological agents such as doxorubicin, cisplatin, and serine/threonine-protein kinase B-Raf (BRAF) inhibitors are associated with worsening renal function. Additionally, Lenvatinib, a tyrosine kinase inhibitor, can produce worsening proteinuria.

## Key points

The rates of thyroid cancer amongst patients with CKD/ESRD are similar to the general population.
Patients with PTC and ESRD may have higher rate of aggressive characteristics compared to patient with PTC and normal renal function [34]. Further research is required to determine a mechanistic link for this association.
There is a higher prevalence of thyroid nodules amongst female patients with ESRD compared to female patients with normal renal function [40].

## Iodine-131 therapy for thyroid cancer and nephrology

Differentiated thyroid cancer is usually treated with partial or total thyroidectomy with possible lymph node dissection. For intermediate-risk or high-risk tumors, radio-iodine 131 ablative therapy is recommended for incomplete resection and to decrease tumor recurrence and mortality [43]. The radio-iodine 131 isotope is a β- and γ-particle emitter with a half-life of approximately 8 days [44]. Twenty percent of blood iodine is absorbed by the thyroid tissue after oral ingestion of radio-iodine 131 [45]. The rest is cleared primarily through the urine, up to 75 % [45]. To date, there are sparse studies that have explored the management of radio-iodine 131 therapy among patients undergoing HD. Therefore, there are no official clinical guidelines. There are a few single site nuclear medicine experiences with this patient population which may be used as a guide [45].

Patients undergoing HD who are receiving radioiodine 131 face two challenging situations. First, due to the prolonged half-life of the isotope, because of limited urinary iodine excretion among patients with renal failure, close family members and caregivers are at an increased risk of radiation exposure. Secondly, the accessibility to a well-equipped facility to perform dialysis service in a well-protected radiation unit post-radioiodine 131 administration is limited [46]. A multidisciplinary team engaging specialists from nuclear medicine, nephrology and endocrinology is crucial to coordinate an optimal treatment plan for these patients [46].

Two conventional methods to determine the dose of radioiodine 131 in patients with thyroid cancer are either to administer an empiric dose or to administer a patient-specific dose utilizing dosimetry. However, the procedure for individual dosimetry is burdensome. Dosimetry calculates the maximum tolerable dose of radioiodine 131 by taking into consideration multiple variables including thyroid remnant volume, metastases, renal clearance, TSH level, and dialysis schedule, assuming no changes in variables occur between dosimetry and therapy.

Interestingly, the actual half-life of radio-iodine 131 was calculated to be 4.5 times higher in patients on HD compared to patients with normal renal function [46]. Most published literature recommends that the treatment dose of radio-iodine 131 for a patient with thyroid cancer on HD should be 13 % to 28 % lower than the standard empirical dose of radio-iodine 131 for a patient with normal renal function [46], [47]. Conversely, in two case reports the authors advocated for increasing the dose of radio-iodine amongst patients with ESRD due to faster clearance rates during dialysis sessions [48].

The standard TH withdrawal for at least four weeks prior to an optimal radioiodine 131 uptake by the thyroid is associated with side effects such as fatigue, headache, and diarrhea. In patients undergoing HD, a single injection of thyrotropin alfa performed 48 h prior to treatment to avoid an excessive TSH elevation was the preferred method and comparable to the two injections of 0.9 mg spread out 24 h apart in patients with normal kidney function [45].

Vermandel et al. proposed a protocol that included inpatient hospital admission for five days to permit dosimetry studies and dialysis in shielded rooms of the nuclear medicine department [45]. On admission to the nuclear medicine department after getting dialysis in the HD area, the administration of radioiodine 131 capsule was performed. Radioiodine 131 whole-body scan was performed three days after treatment. First and second dialysis was performed at 42 h and 90 h, respectively, after the administration of the radioiodine 131 capsule using a portable dialysis machine in the nuclear medicine department. The dialysates were directly expelled through connections to the decay tanks prior to being freed into the public sewage treatment system after four to five months of decay [45]. The paucity of thyroid cancer in patients undergoing HD contributes to the limited data in the current literature. Thus, reaching an official consensus regarding optimal management of these patients is challenging.

## Key points

Patient with ESRD on HD undergoing radioiodine 131 treatment for thyroid cancer are at increased risk of prolonged radiation transmission risk due to limited urinary iodine excretion.
There is no consensus guideline for the dosing and timing of radioiodine 131 amongst patients with ESRD. However, there are some key clinical considerations.
  The half-life of radioiodine 131 can be 4.5 times higher in patients on HD compared to patients with normal renal function [46].
  There is conflicting data regarding using increased or decreased empiric radioiodine 131 doses in patients with ESRD on HD and thyroid cancer [46], [47], [48]. Most research recommends using a lower empiric dose of radioiodine 131.
  Dosimetry studies are an optimal way to determine patient-specific radioiodine 131 doses among patients with ESRD on HD and thyroid cancer. However, this resource is limited.
A multidisciplinary team engaging specialists from nuclear medicine, nephrology and endocrinology is best used to coordinate and optimized radioiodine 131 therapy for patients with ESRD on HD and thyroid cancer [46].
Further research is required to establish the metabolism, efficacy, and excretion of radioiodine 131 amongst patients with ESRD on HD.

## Conclusion

In summary, patients with ESRD have a higher prevalence of subclinical hypothyroidism. While the mechanistic link between thyroid and kidney is not well understood, increasing evidence points to a bi-directional connection between hypothyroidism and CKD. Untreated hypothyroidism in patients with renal failure has been associated with worsening renal eGFR. Treatment of hyperthyroidism in patients with normal renal function may unmask unknown CKD. Additionally, untreated hyperthyroidism accelerates CKD significantly. The prevalence of thyroid cancer in patients with CKD is similar to the general population. Papillary thyroid cancer cases in patients with ESRD have a significantly higher rate of aggressive characteristics. There is a compelling need for further research on how to best manage thyroid cancer radioiodine 131 therapy among patients with renal failure. Most published literature recommends that the treatment dose of radioiodine 131 for a patient with thyroid cancer on hemodialysis should be decreased by 13 % to 28 % [46], [47]. There is a need to develop guidelines regarding dosing and timing of thyroid cancer radioiodine 131 ablation therapy among this key group of patients.

## CRediT authorship contribution statement

**Joe M. Chehade:** Writing – review & editing, Writing – original draft, Visualization, Conceptualization. **Heiba F. Belal:** .

## Declaration of competing interest

The authors declare that they have no known competing financial interests or personal relationships that could have appeared to influence the work reported in this paper.
